# Effects of mental fatigue on risk preference and feedback processing in risk decision-making

**DOI:** 10.1038/s41598-022-14682-0

**Published:** 2022-06-23

**Authors:** Huiqiao Jia, Chiuhsiang Joe Lin, Eric Min-yang Wang

**Affiliations:** 1 Department of Industrial Management, National Taiwan University of Science and Technology, Taipei, 106335 Taiwan; 2 Institute of Human Factors and Safety Engineering, Hunan Institute of Technology, Hengyang, 421002 People’s Republic of China; 3 Department of Industrial Engineering and Engineering Management, National Tsing Hua University, Hsinchu, 300044 Taiwan

**Keywords:** Psychology, Human behaviour

## Abstract

Mental fatigue is a common phenomenon in modern people, especially after a long period of mental work. Individuals frequently have to make critical decisions when in a mentally fatigued state. As an important and complex cognitive function, risk decision-making might be influenced by mental fatigue, which is consequent with increased distraction and poor information processing. However, how mental fatigue shapes individuals’ decision-making remains relatively unclear. The purpose of this study was to examine the effect of mental fatigue on risk decision-making performance and risk-preference in a simple gambling task, using both behavioral methods and event-related potential techniques. Forty young adults were divided into a mental fatigue group and a no-fatigue group and participated in the experiments. Results showed that individuals with mental fatigue tended to be more risk-averse than those without fatigue when facing risk options. The P300 amplitudes were smaller and FRN amplitudes were larger in the mental fatigue group than in the no-fatigue group. These findings provide insight into a relationship between mental fatigue and risk decision-making, from the perspective of the neurological mechanism.

## Introduction

In modern society, people’s work tends to be more and more mentally demanding, rather than physically demanding. Long hours of mental work can easily lead to mental fatigue. It is not rare that people have to make decisions (sometimes even critical ones) when they are mentally fatigued. For instance, truck drivers often have to make quick decisions in unexpected situations when/after continuously driving (which easily leads to mental fatigue)^1^. In many complex cases, surgeons sometimes have no choice but to perform surgeries lasting many hours and make medical decisions^2^.

Mental fatigue is a complex phenomenon thus it is really hard to define and describe it in scientific terms. In the present study, we adopt the definition by Boksem’s study and described mental fatigue as a state of individuals that occurs during or after a prolonged period of cognitive activity, and it is always accompanied by declines in the capacity and efficiency of mental activities^3^. Mental fatigue is observed to impair selective attention^4^, weaken cognitive control^5^, and decrease high-level information processing^6^ or even decline physical performance^7^. ﻿These changes may combine to impair the ability to make favorable decisions under conditions of risk.

Previous studies have shown that decision-making can be affected by mental fatigue in different fields and conditions. Researchers found mental workload disturbs the processing of risk decision-making^8^, and individuals with higher workloads are more inclined to make conservative choices^9^. One study observed that the parole decision by one parole board tends to be more conservative after a prolonged time working, which indicated mental fatigue could affect the decision made by the parole board^10^. People become more risk-averse after taking tasks that need many mental resources^11^. On the contrary, other researchers claimed that mentally fatigued participants have higher levels of sensation seeking and are more willing to take risks than non-fatigued participants^12^. Also, a tendency toward making risky decisions was observed in a population with sleep deprivation (always accompanied by a fatigued state)^13^. Although the results are mixed, these shreds of evidence indicate that the decision ability and quality of fatigued decision-makers are likely to be affected by their mental state. Thus, clarifying the relationship between mental fatigue and risk decision-making remains an important issue.

The ability of processing feedback information plays an important role in risk decision-making. The feedback outcome evaluation could modulate individuals’ choices. Research has demonstrated that, in many cases, individual differences in risk-taking are driven by differences in the perception and evaluation of risks and returns^14^. With exquisite temporal resolution, event-related potentials (ERPs) have advantages for investigating the dynamic mechanisms of cognitive processes, especially the stage of outcome evaluation. Many previous studies have used ERPs to investigate risk decision-making^15^. Two ERP components, P300 and feedback-related negativities (FRN) are particularly sensitive to the processing of outcome evaluation and often used to examine cognitive processes correlated with risk decision-making and feedback-evaluation processing^16,17^. Studies have demonstrated that P300 and FRN carry important information on feedback processing. Specifically, the P300 reflects a later, top-down controlled feedback evaluation process in decision-making^18^, whereas FRN may reflect the neural processes that differentiate favorable outcomes from unfavorable outcomes^19^.

P300, which is a centroparietal positivity that is often associated with the allocation of cognitive resources. Larger P300 amplitudes indicate that more resources are allocated to the ongoing task^20^. Numerous researchers have demonstrated the P300 component is reduced by the increase of mental fatigue/ mental workload^21,22^. It implies that P300 could be an index of impaired cognitive activities caused by mental fatigue. Combined with the fact that P300 is sensitive to feedback processing, it indicates mental fatigue could be an important factor that affects the feedback processing during risk decision-making.

FRN, which is maximal at frontocentral scalp sites and is supposed to originate in the anterior cingulate cortex (ACC)^23^. Larger FRN amplitudes also indicate a stronger motivational impact of the current event^23,24^. Evidence from neuroimaging and event-related potential studies has shown that mental fatigue alters activity in the anterior cingulate cortex (ACC) and the striatum^5^ and reduces functional connectivity between the emotion-related brain regions (e.g., the amygdala) and the prefrontal cortex^25^, suggesting that cognitive control is less recruited during fatigue. Thus, when mentally fatigued, it is possible that one’s feedback processing that requires the neural activity of the ACC may be compromised.

To our knowledge, however, there are as yet no ERP studies directly comparing electrophysiological responses of feedback processing between mental fatigue individuals and no fatigue individuals. We, therefore, sought to investigate whether mental fatigue modulates both the behavioral performance and electrophysiological activity of feedback processing using a monetary gambling task in the present study. In light of the proposal that mental fatigue might influence risk decision-making, and the facts that P300 and FRN might vary according to different mental states, we hypothesized that (1) risk preference differed between mental fatigue individuals and no fatigue individuals, (2) mental fatigue individuals showed lower P300 than their counterparts, (3) FRN amplitudes differed between the two groups. Given the important implications of FRN and P300 to feedback processing in risk decision-making, we also assumed that ERP components were correlated with risk preference.

## Methods

### Participants

Forty-two Han Chinese students were recruited from the Hunan Institute of Technology, and two participants were excluded due to excessive artifacts in their EEG recordings. ﻿All participants were right-hand dominant and had a normal or corrected-to-normal vision and no known neurological disorders. None of the participants worked night shifts or used prescription medications. Participants were requested to have at least 8 h of sleep the night and avoid strenuous physical and mental activities as much as possible before they came to join the experiment. The forty participants (mean age = 20.35 ± 1.58 years) were evenly divided into two groups that differed in the mental fatigue manipulation. Participants’ demographic information is presented in Table 1. The control group included 13 males and 7 females, while the fatigue group included 12 males and 8 females. There was no significant age (t = 0.396, p = 0.694 > 0.05) difference between the two groups. Participants gave informed consent before the experiment and received monetary compensation upon completion of the study. The experimental protocol was conducted following the Declaration of Helsinki and was approved by the local Ethics Committee (Hunan Institute of Technology, Hengyang, China).

**Table 1:** Participants’ demographic information.

Demographic information	Mental fatigue	No fatigue	t	P
Number (n)	20	20	–	–
Male (n)	12	13	–	–
Age (years)	20.45 ± 1.54	20.25 ± 1.65	0.396	0.694

### Materials

#### Fatigue manipulation

**Figure 1:**
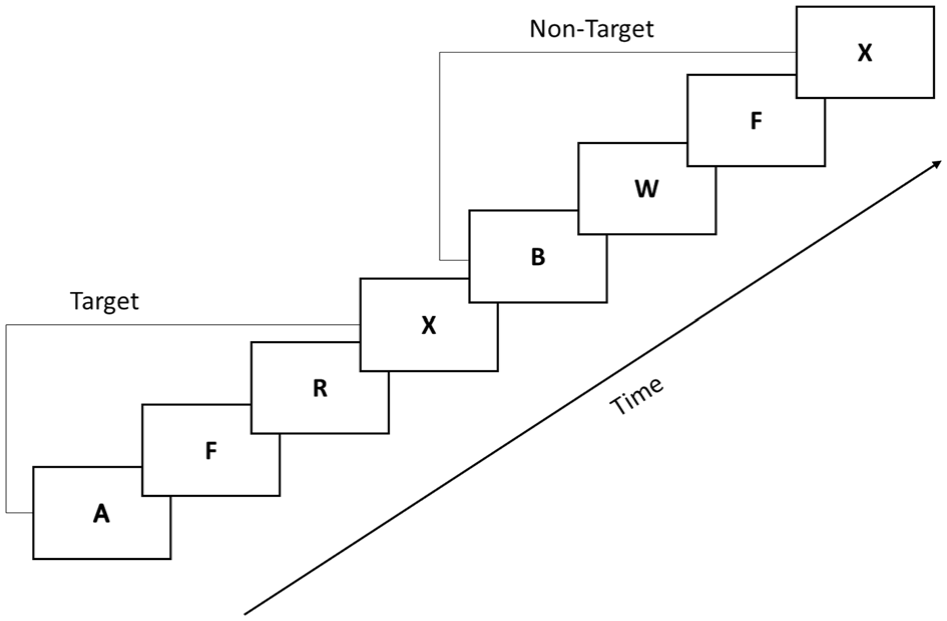
AX-CPT paradigm.

In accordance with earlier studies^7,26^, we used the AX-CPT task to induce mental fatigue, as shown in Fig. 1. In the task, sequences of letters were presented on the center of the computer screen. Participants were instructed to press the right button of the mouse in response to an appearance of the letter “X” following a letter “A” (target trials). Otherwise, they were required to press the left button of the mouse. The mental fatigue group was asked to perform AX-CPT for 60 min. This fatigue-induced experimental task involves working memory and attention, which require a continuous input of cognitive resources. After a sustained task process, individuals will experience strong fatigue^26^.

#### Subjective measurement

Subjective mental fatigue was measured using the Fatigue Scale (FS-14) developed by Chalder^27^. The FS-14 is a questionnaire with 14 items divided into mental and physical subscales. Each of the items is rated on a four-point Likert-type scale (0, 1, 2, 3) of 'better than usual' (0), 'no more than usual ' (1), 'worse than usual' (2), 'much worse than usual ' (3). All participants were required to complete the questionnaire both before and after the experimental session. The internal consistency of the questionnaire is 0.89.

#### Risk decision tasks

**Figure 2:**
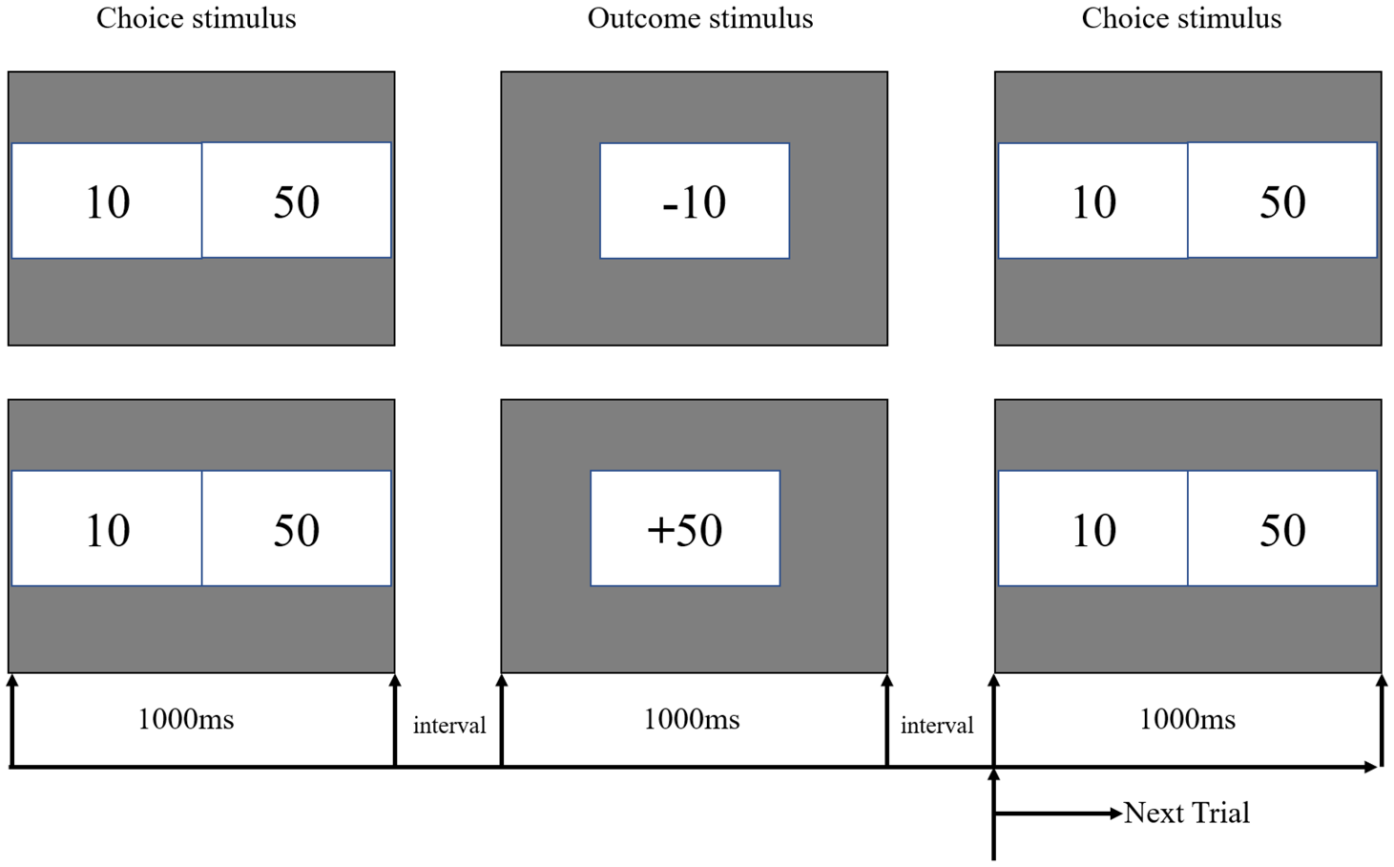
Single outcome gambling task.

The Single Outcome Gambling Task (SOG) was used as the risk decision task, which could examine the risk-taking of participants during decision-making. The paradigm is shown in Fig. 2. The SOG task requires participants to make choices between one of two boxes labeled “10” or “50”, each representing a monetary value. After the choice is made, feedback is provided to show if the participant got a win or loss and its value. Selecting the option with higher monetary value (the box labeled “50”) is considered to be riskier.

### Procedures

All the experiments were conducted in the Human Factors Engineering Laboratory at the Hunan Institute of Technology. Participants were divided into two groups: a mental fatigue group and a control (no fatigue) group, in which the participants would not experience mental fatigue. The twenty subjects in the mental fatigue group were randomly selected to do a mental fatigue-inducing task for 60 min and to complete the fatigue self-assessment scale (FS-14). Another twenty subjects were asked to fill in the scale directly.

Before the formal task, all participants were told that they would take part in a monetary gambling game. They were informed of the instructions of the game as well as the ERP procedures. Participants were encouraged to respond in a way that could maximize the total score; in other words, they were to try to earn as much money as possible. Earning higher scores would lead to them receiving more money after the experiment.

During the experiment, the participants were seated in a comfortable chair that was 100 cm in front of a computer screen in a sound-isolated, dimly lit room. The participants were required to complete a total of 192 trials of the gambling task. A single trial of the gambling task began with a fixation cross appearing on the screen center for 1000 ms (ms). After that, two boxes were presented in the center of the screen. The left-hand box was labeled 10 and the right one was labeled 50. The numbers 10 and 50 indicated the amount of a bet: 10 and 50 Jiao RMB, that is, approximately 20 and 80 US cents, respectively. The options remained on the screen until the participants selected one of the numbers by pressing the F or J key on the keyboard with their left or right index finger (F for the alternative on the left and J for the one on the right). After that, the result of the participants' choice appeared with a “+” or “−” symbol, which indicated that the participants won or lost 10 or 50 Jiao RMB, respectively (see Fig. 2). The feedback display remained visible for 1500 ms. Then a black screen was presented for a short interval (varying randomly between 700 and 1200 ms) before the beginning of the next trial. We used E-prime 2.0 to present the stimuli and collect participants’ behavioral responses.

### EEG recording and processing

Electroencephalogram (EEG) data were recorded from 64 scalp sites using an elastic cap with sintered Ag/AgCl electrodes according to the Extended International System sites frontal lobes. Horizontal electrooculogram (HEOG) data were recorded from electrodes placed at the outer canthi of both eyes. Vertical electrooculogram (VEOG) data were recorded from electrodes placed above and below the left eye. All electrode impedances were maintained below 5 kΩ. EEG and EOG signals were continuously sampled at 1000 Hz with a bandpass filter of 0.01 to 100 Hz.

Data were preprocessed in Matlab R2021a (MathWorks, Natick, USA). The recorded EEG data were filtered with a 0.01–30 Hz finite impulse response filter with zero phase distortion. Filtered data were segmented, beginning at 200 ms before the onset of the outcome and lasting for 1200 ms. All epochs were baseline-corrected concerning the mean voltage over the 200 ms preceding the onset of the outcome, followed by averaging in the association with four different feedback conditions (+ 10, − 10, + 50, − 50).

In this study, two ERP components, namely, FRN and P300, were chosen for analysis. ERPs were averaged from the EEG data. Previous research has reported that FRN is maximal in the fronto-central area of the scalp, whereas P300 is maximal in the centro-parietal area. We focused on FRN and P300 at the Fz, Cz, and Pz electrodes because previous works suggested that these components were largest in these electrodes^23,28,29^. Based on a previous study^16^ and visual inspection, the FRN amplitude was measured as the mean amplitude within a 240–350 ms time window, while the P300 amplitude was measured as the mean amplitude within a 320–400 ms time window.

### Data analysis

This study focused on the effect of mental fatigue on subsequent outcomes in decision-making. The analyses of electrophysiological data focused on three factors: outcome valence, outcome magnitude, and mental fatigue condition. Outcome valence refers to the valence of the outcome (win vs. lose) of the current trial. Outcome magnitude refers to the magnitude of the outcome (small vs. large), which in this study depended upon participants' choices (low-risk vs. high-risk) in the current trials.

Participants’ ages, decision times, and risk ratio were analyzed using independent samples t-tests. FRN and P300 amplitudes were analyzed using repeated-measures analysis of variance (RMANOVA) with outcome valence and outcome magnitude treated as the within-subjects variables, and group type was treated as a between-subjects variable.

Statistical analysis was performed in SPSS Statistics 25.0 (IBM, Somers, USA). For all the analyses listed in this study, the significance level was set at 0.05. A Greenhouse–Geisser correction for ANOVA tests was used when appropriate. Partial eta-squared (η^2^_p_) was used to demonstrate the effect size in the ANOVA tests, where 0.05 represented a small effect, 0.10 indicated a medium effect, and 0.20 represented a large effect^30^.

## Results

### Subjective ratings

The mean score on the mental symptom subscale of the Fatigue Scale was 4.05 ± 1.36 in the fatigue group, while it was 1.95 ± 1.47 in the control group. The internal consistency value of FS-14 scale was 0.986 in control group, while it was 0.993 in fatigue group. An independent-samples t-test was conducted on the Fatigue Scale scores of the fatigue group and the no-fatigue group. Mean scores were significantly higher in the mental fatigue group than in the no-fatigue group (t = 4.699, p = 0.000 < 0.01), revealing that the fatigue manipulation was successful. The results indicated that the mental fatigue induction process was effective and the grouping was reasonable.

### Behavioral results

#### Decision time

Table 2 shows the behavioral results and risk choices of the two groups. The average decision time of the participants with mental fatigue was 531.03 ± 76.87 ms, while it was 525.93 ± 52.20 ms in the participants without fatigue. The internal consistency value of decision-making time was 0.805 in control group, while it was 0.741 in fatigue group. An independent sample t-test conducted on the decision times of the mental and the no-fatigue groups (t = 0.245, p = 0.808 > 0.05) indicated that there was no significant difference in average decision times between the two groups.

**Table 2:** Behavioral results and risk preference.

	Mental fatigue	No fatigue	t	p
Decision time (ms)	531.03 ± 76.87	525.93 ± 52.20	0.245	0.808
Choose 10 (frequency)	107.6 ± 19.91	95.2 ± 18.40	2.045	0.048*
Choose 50 (frequency)	83.65 ± 19.93	96.25 ± 18.66	− 2.064	0.046*
Risky choice ratio (%)	0.44 ± 0.10	0.50 ± 0.10	− 2.064	0.046*

Statistically significant difference at *p < 0.05, **p < 0.01 and in bold type.

#### Risk preference

In the gambling task, we defined option 50 to be the risky choice, which means high risk and high return. Meanwhile, option 10 represents the risk-avoidant choice. Therefore, the ratio of the subject choosing 10 and 50 was used to measure risk preference. The internal consistency value of risk preference ratio was 0.841 in control group, while it was in 0.764 fatigue group. The independent samples t-test showed that the risk choice ratio of the fatigue group was significantly lower than the no fatigue group (t =  − 2.064, p = 0.046 < 0.05). Therefore, it appeared that mental fatigue had an impact on the risk preference of the individuals when making risk decisions. The mean ratio of risk preference in the fatigue group was 0.44, while it was 0.50 in the non-fatigue group, indicating that the participants with mental fatigue tended to make more conservative choices than participants with no fatigue in their risk choices.

### EEG results

**Figure 3:**
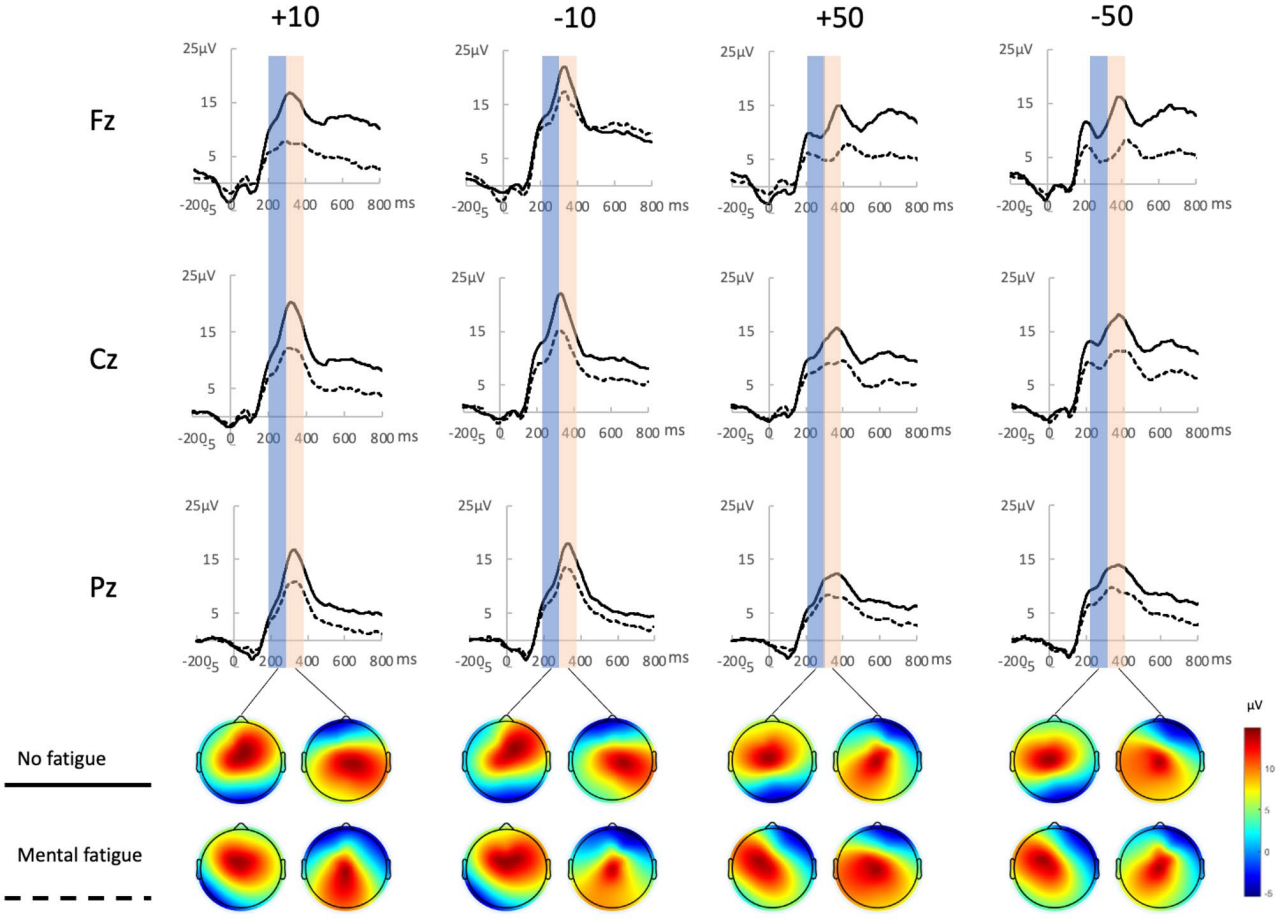
Grand average ERP waveforms and Topographic scalp maps elicited by four different outcome stimuli at the electrode sites Fz, Cz, and Pz.

Figure 3 shows the grand average ERP waveforms elicited by four different outcome stimuli (+ 10, − 10, + 50, − 50) at the electrode sites Fz, Cz, and Pz. The ﻿differences between the two groups in four feedback conditions were shown by Topographic scalp maps depicted in Fig. 3. Repeated-measures ANOVA results are compiled in Table 3.

**Table 3:** Mixed-model ANOVA results.

Variables or interactions	P300				FRN			
	Amplitude		Latency		Amplitude		Latency	
	F	p	F	p	F	p	F	p
Group	**10.753**	0.002*	0.109	0.743	**13.092**	0.001**	**6.109**	0.018*
Valence	1.322	0.257	**48.954**	0.000**	**67.148**	0.000**	**40.996**	0.000**
Magnitude	**14.129**	0.001**	1.159	0.288	4.109	0.050	0.074	0.786
Group × Valence	0.220	0.642	0.021	0.885	3.799	0.059	0.172	0.681
Group × Magnitude	0.000	0.987	0.125	0.726	0.003	0.958	0.632	0.432
Valence × Magnitude	0.077	0.783	3.097	0.087	0.037	0.849	2.698	0.109
Group × Valence × Magnitude	1.961	0.169	0.366	0.549	2.166	0.149	0.189	0.666

Statistically significant difference at *p < 0.05, **p < 0.01 and in bold type.

#### P300

As is shown in Table 3, P300 amplitudes with RMANOVA revealed a main effect of fatigue difference and feedback magnitude. P300 amplitudes of mental group is smaller than no fatigue group [(8.803 ± 0.989) μV vs. (13.390 ± 0.989) μV, F (1,38) = 10.753, p < 0.01, η^2^ = 0.221]. The internal consistency value of P300 amplitudes was 0.785 in mental fatigue group, while it was 0.722 in fatigue group. P300 amplitudes of large magnitude (50) is larger than small magnitude (10) [(12.010 ± 0.752) μV vs. (10.183 ± 0.728) μV, F (1,38) = 14.129, p < 0.01, η^2^ = 0.271].

Analysis of P300 latency with RMANOVA revealed a main effect of feedback valence. P300 latency of “lose” condition is significant longer than “win” condition [(358.581 ± 4.207) ms vs. (353.113 ± 5.047) ms, F(1,38) = 48.954, p < 0.01, η^2^ = 0.563]. No other significant effects or interactions were observed.

#### FRN

Analysis of FRN amplitudes revealed significant effects of fatigue difference and feedback valence. FRN amplitudes of mental group is larger than no fatigue group [(8.112 ± 1.112) μV vs. (13.802 ± 1.112) μV, F (1,38) = 13.092, p < 0.01, η^2^ = 0.256]. The internal consistency value of FRN amplitudes was 0.887 in mental fatigue group, while it was 0.940 in the control group. FRN amplitudes of “lose” is larger than “win” [(9.471 ± 0.780) μV vs. (12.443 ± 0.833) μV, F (1,38) = 67.148, p < 0.01, η^2^ = 0.639]. Note that since FRN is a relative negative going component, ‘larger FRN’ means this component is more negative.

Analysis of FRN latency with RMANOVA also revealed a main effect of mental fatigue and feedback valence. FRN latency of mental fatigue group is longer than no fatigue group [(270.956 ± 5.008) ms vs. (253.450 ± 5.008) ms, F (1, 38) = 6.109, p < 0.05, η^2^ = 0.138]. FRN latency of lose condition is longer than win condition [(274.119 ± 3.767) ms vs. (250.288 ± 4.222) ms, F (1,38) = 40.996, p < 0.01, η^2^ = 0.519]. Repeated-measures ANOVAs revealed no significant interactions.

### Correlation analyses

Pearson's correlation analyses revealed that P3 amplitudes after some conditions were positively correlated with the risk ratio in small magnitude (10) in mental fatigue individuals (Table 4). Additionally, the FRN amplitudes evoked by various conditions were negatively correlated to the mental fatigue scores except for large-magnitude gain (50).

**Table 4:** Correlation analysis results.

	The risk ratio of the mental fatigue group	
	r	p
P300 + 10	**0.463**	0.040**
P300−10	**0.444**	0.050*
P300 + 50	0.179	0.450
P300−50	0.379	0.100
FRN + 10	**− 0.570**	0.009**
FRN−10	**− 0.559**	0.010**
FRN + 50	0.292	0.211
FRN−50	**− 0.543**	0.013**

Correlation coefficient is statistically significant at *p < 0.05, **p < 0.01 and in bold type (all p-values were uncorrected).

## Discussion

The primary purpose of this study was to investigate whether mental fatigue affects risk decision-making, which we intended to measure with both behavioral and brain activities. As an experimental manipulation, the fatigue group performed an AX-CPT task before the decision task. The success of the manipulation is demonstrated by the significant increase of mental fatigue scores compared to the no fatigue group. Both of the two groups performed a simple monetary gambling task in which the risk preferences were revealed and the brain activities were recorded.

The average decision time showed that there was no significant difference between individuals with and without mental fatigue. As is known and supported by previous studies, fatigued individuals have difficulties in concentration, accompanied by drowsiness and low efficiency in performance^6,31–34^. These states lead to a reduced capacity to respond. However, the decision response time results in this study did not show significant differences between the fatigue and no-fatigue groups, which indicated that mental fatigue did not have an impact on the decision time in the current risk decision-making task. This lack of an effect of mental fatigue on decision time might be explained as follows. First, in the process of the risk decision-making task, participants tended to react as quickly as they could so as not to exceed the trial time. This time pressure may have led to the similarly short decision times in the two groups. Second, the basic cognitive and operational capabilities of the subjects in the two groups were similar, further explaining the absence of a great difference in the choices and judgments on the decision-making tasks, as well as in the reaction times of decision-making.

Compared to the control group, participants with mental fatigue tended to choose more low-risk and low return options (option 10) rather than high risk and high return options (option 50). Results of risk choice ratio revealed that individuals with mental fatigue made more conservative choices, which indicates that they are more risk-averse. One potential interpretation for these findings is that mental fatigue may change the sensitivity to outcomes, and analyzing the current situation and making a subsequent decision may also require more cognitive resources, so individuals may wish to avoid risk-taking. Therefore, in a state of mental fatigue, individuals might be more inclined to choose conservative options in the process of risk decision-making. A previous study pointed out that when the cognitive ability is low or the cognitive load is high, individuals are more inclined to adopt conservative strategies^9^. Wang et al. also found a similar phenomenon; individuals tend to make more conservative choices when mentally fatigued than when not^35^. Although different observations show a tendency toward making risky decisions in a population with sleep deprivation^13^ and individuals with ego depletion^12^, they focused on different mental conditions. To some extent, sleep deprivation is significantly different from general mental fatigue caused by prolonged mental activity. Sleep deprivation is a more complex and extreme condition. It can impair not only cognitive functions but also physical functions^36^. The participants in this study were healthy college students who had rested sufficiently before the experiment. In addition, although ego depletion is related to mental fatigue, there is no evidence that they are equal in mental state. Thus, the contradictory results may be ascribable to the different conditions and populations. One possible limitation of this study is that the gambling task we used as the decision task is likely not specifically just measuring risk taking. Risk taking behaviors in monetary paradigms might be influenced not only by the amount of money, but also the probability of the win/lose. The risk choice ratio in gambling task might partially reveal the risk preference of individuals. Hence, in further studies, we will consider more factors such as the probability of win/lose setting in the task.

We found a reduced P300 in mentally fatigued individuals compared to no fatigue individuals, which provided evidence for a lack of cognitive resources in mentally fatigued people. Our findings are in line with previous reports about the ERP shift of P300 in mental fatigue individuals. Previous reports have likewise described reduced P300 amplitudes in individuals with mental fatigue compared to no fatigue ones^22,37,38^. Our study showed that the P300 amplitude of the mental fatigue group was significantly smaller than the no fatigue group. This might indicate that individuals with mental fatigue did not have an equal ability to adjust their behaviors to make better decisions. In addition, the P300 wave component has been reported to play an important role in enabling individuals to differentiate good from bad outcomes during decision-making and thereby allowing them to optimize their actions^39^. In the current study, P300 amplitude is seen to be positively correlated to risk preference in mental fatigue individuals. From the relationship between P300 and risk preference, we can also suggest that the reduced P300 amplitude is related to risk-averse. This is consistent with our findings in behavioral results that mentally fatigued individuals tend to be more risk avoidance than their counterparts. It suggested that P300 amplitude could be an electrophysiological index to risk-taking when mental fatigue.

FRN is closely related to the ACC (a structure of central importance in the processing of reward, punishment, and effort demands)^23^. Many studies have demonstrated FRN component is closely related to risk-taking in decision making^23,40–42^. Individual differences in FRN amplitude have been related to risk-taking^43,44^ In this study, we found that FRN amplitude was significantly larger (more negative) in the mental fatigue group than the no-fatigue group. Combined with the risk-averse tendency that we found in the mental fatigue group, we might claim that larger FRN amplitude is related to risk-averse. In an ERP study, lower FRN amplitude is shown in risk-seeking than risk-averse individuals. This is partially consistent with what we found in the present study^44^. On the other hand, higher punishment sensitivity was associated with larger FRN amplitude^45,46^. It indicates that mental fatigue might lead to higher punishment sensitivity, which results in a risk-averse. In terms of the influence on the magnitude selected, the results showed a significant difference in the FRN amplitudes between the different magnitudes (10 and 50). This also implies differences in the cognitive mechanism of the subjects' brainwaves when they made different choice magnitudes during risk decision-making. One explanation of these results could be that mental fatigue affects the individual’s risk tolerance when the options are risky. The correlation analyses revealed that FRN amplitude is negatively correlated to the risk ratio in the mental fatigue group, which means the larger amplitude is related to risk avoidance. These pieces of evidence also support our findings in the behavioral domain.

The present study provides insights into the electrophysiological processing of differential responses to reward and punishment between mental fatigue individuals and no fatigue individuals. This study demonstrates a decreased P300 amplitude following a loss as well as increased FRN responses in mental fatigue individuals. These findings suggest an underlying deficit in feedback processing, which may increase the propensity to be more risk-averse in mental fatigue individuals.

To sum up, by examining behavioral responses and brainwave characteristics, the current study investigated whether mental fatigue affects the sensitivity to outcomes and risk preferences of individuals. Indeed, the results of the present study revealed that the risk preference and ERP components were both influenced by mental fatigue. Individuals with mental fatigue tended to make conservative choices during decision-making. According to the ERP results, this phenomenon might be explained by mental fatigue disturbing the processing of decision-making, especially the feedback processing of outcomes, which is considered to be one of the significant factors in decision-making.

## Conclusion

The present study demonstrates the effect of mental fatigue on risk decision-making. Individuals with mental fatigue may have lower risk tolerance and consequently be risk averse. Our research contributes to the understanding of the effects of fatigue on decision-making by showing that mental fatigue affects both the risk preferences and the processing of feedback. These findings provide insights into the electro-physiological processing during risk decision-making and may have practical implications for making appropriate decisions when in different mental states.
